# A Metabolomics Study of Feces Revealed That a Disturbance of Selenium-Centered Metabolic Bioprocess Was Involved in Kashin–Beck Disease, an Osteoarthropathy Endemic to China

**DOI:** 10.3390/nu15214651

**Published:** 2023-11-02

**Authors:** Yan Wen, Bingyi Wang, Panxing Shi, Xiaoge Chu, Sirong Shi, Yao Yao, Lu Zhang, Feng Zhang

**Affiliations:** 1 Key Laboratory of Trace Elements and Endemic Diseases of National Health and Family Planning Commission, School of Public Health, Health Science Center, Xi’an Jiaotong University, Xi’an 710061, China; bingyiwang@stu.xjtu.edu.cn (B.W.); spxing0813@stu.xjtu.edu.cn (P.S.); 3121315054@stu.xjtu.edu.cn (X.C.); Shisirong@stu.xjtu.edu.cn (S.S.); yyxjtu@xjtufh.edu.cn (Y.Y.); snnuyang@snnu.edu.cn (L.Z.); fzhxjtu@mail.xjtu.edu.cn (F.Z.); 2 Key Laboratory for Tumor Precision Medicine of Shaanxi Province, Department of Endocrinology, The First Affiliated Hospital of Xi’an Jiaotong University, Xi’an 710061, China; 3 Medical Department, The First Affiliated Hospital of Air Force Medical University, Xi’an 710032, China

**Keywords:** Kashin–Beck disease (KBD), metabolomics, intestine, selenium, lipid metabolism, bile acids and salts, free fatty acid

## Abstract

**Background**: Kashin–Beck disease (KBD) is a distinct osteoarthropathy in China with an unclear pathogenesis. This study aims to explore whether perturbations in the intestine metabolome could be linked to KBD individuals. **Methods**: An investigation was conducted in KBD endemic villages and fecal samples were collected. After applying inclusion and exclusion criteria, a total of 75 subjects were enrolled for this study, including 46 KBD (including 19 Grade I KBD and 27 Grade II KBD) and 29 controls. Untargeted metabolomics analysis was performed on the platform of UHPLC-MS. PLS-DA and OPLS-DA were conducted to compare the groups and identify the differential metabolites (DMs). Pathway analysis was conducted on MPaLA platform to explore the functional implication of the DMs. **Results:** Metabolomics analysis showed that compared with the control group, KBD individuals have a total of 584 differential metabolites with dysregulated levels such as adrenic acid (log_2_FC = −1.87, VIP = 4.84, *p* = 7.63 × 10^−7^), hydrogen phosphate (log_2_FC = −2.57, VIP = 1.27, *p* = 1.02 × 10^−3^), taurochenodeoxycholic acid (VIP = 1.16, log_2_FC = −3.24, *p* = 0.03), prostaglandin E3 (VIP = 1.17, log_2_FC = 2.67, *p* = 5.61 × 10^−4^), etc. Pathway analysis revealed several significantly perturbed pathways associated with KBD such as selenium micronutrient network (Q value = 3.11 × 10^−3^, Wikipathways), metabolism of lipids (Q value = 8.43 × 10^−4^, Reactome), free fatty acid receptors (Q value = 3.99 × 10^−3^, Reactome), and recycling of bile acids and salts (Q value = 2.98 × 10^−3^, Reactome). Subgroup comparisons found a total of 267 differential metabolites were shared by KBD vs. control, KBD II vs. control, and KBD I vs. control, while little difference was found between KBD II and KBD I (only one differential metabolite detected). **Conclusions:** KBD individuals showed distinct metabolic features characterized by perturbations in lipid metabolism and selenium-related bioprocesses. Our findings suggest that the loss of nutrients metabolism balance in intestine was involved in KBD pathogenesis. Linking the nutrients metabolism (especially selenium and lipid) to KBD cartilage damage should be a future direction of KBD study.

## 1. Introduction

Kashin–Beck disease (KBD) is a kind of osteoarthropathy endemic to China and is characterized by enlarged, deformed, and painful joints at multiple locations. The initial pathological changes in KBD occur to the epiphyseal cartilage and articular cartilage. Chondrocyte necrosis is pronounced in the deep zone of KBD cartilage and clusters at several focal areas. Such alteration starts as early as childhood or adolescence. The symptoms worsen with age. Secondary osteoarthritis phenotype becomes prominent in adult KBD patients, including narrowness of joint space, painful joints, osteophytes, etc. [1].

The disease distributes extensively in China, in a belt-like region ranging from northeast to southwest China. Such specific geographic distribution indicates its close relation to the local environment, which is proven by mounting studies [2]. Biogeochemical hypotheses reckon that the distinct chemical background (mainly selenium deficiency) in KBD areas results in its epidemic. Inadequate intake of selenium is considered a key risk factor; this has obtained a lot of support from population, animal, and cell-based evidence [2,3,4,5,6,7,8]. Selenium supplementation has been used to prevent and control KBD in some regions of China, which has proven to be effective [9]. One hypothesis suggests that grain contamination by certain fungi toxins causes the damage of KBD [2]. Wang Kewei et al. concluded that KBD is primarily food sourced in etiology [10]. Recent research has also revealed that genetic components are involved in KBD [11]. Several genetic variants have been found to be associated with KBD [12]. However, the complicated pathogenesis of KBD remains unclear until now.

Metabolites refers to low-molecular-weight compounds produced as intermediate or end products of metabolic processes in organisms, as well as their derivatives. Metabolomics technique facilitates the measurement of abundant metabolites in certain biological unites at one time, which are informative in elucidating the biological process under specific circumstances. They are valuable in the study of pathogenesis, diagnosis, and treatment of diseases such as osteoarthropathy. For example, Wu et al. concluded that the lack of balance in central carbon energy metabolism has significant implications in OA progression [13]. Those findings drive people to investigate the possible role of metabolites in KBD development. A previous study found a distinct signature of metabolites especially the dysregulation of lipid metabolism in KBD serum [14]. Fecal metabolites are a series of final products from cellular and microbial metabolic processes in the human intestinal tract, which could be shaped and affected by external exposure from diet. They can be absorbed into the circulation and exert an influence on individual’s skeleton health [15]. For example, a previous research revealed that CAT, as a metabolite of intestinal tract, has a protective effect against osteoarthritis via the inhibition of HIF-1α and activation of SLC2A1 [16]. These clues suggest a potential role of fecal metabolites in the mechanism of cartilage damage in KBD under exposure to risky environmental factors. In summary, it is necessary to further explore the fecal metabolite profile of KBD for deepening the understanding of KBD’s mechanism as well as other osteoarthtopathy.

## 2. Material and Methods

### 2.1. Study Population Recruitment and Sample Collection

All study subjects were recruited from Linyou city in Shaanxi province, a typical KBD area in central China. We informed the residents about the survey in advance and took some time to explain the aim of this study. After the subject approved to participate, a questionnaire survey was made to collect demographic information including age, sex, BMI, residential location, etc. Meanwhile, clinical symptom examination was conducted and X-ray inspections were made at the knee, hand, and ankle joints. KBD was diagnosed according to the classic criteria of China (WS/T 207-2010) by two independent doctors. KBD patients were divided into two grades: Grade I and Grade II. Multiple and symmetrical enlarged joints in the fingers are the typical disease marker of Grade I KBD patients. Additionally, Grade II patients also have fingers shortened. Healthy controls were recruited form the population in the same KBD area. All subjects with a history of other osteoarthtopathy (such as primary osteoarthritis, rheumatoid arthritis, or other genetic bone diseases) or gastrointestinal diseases (such as gastroenteritis, constipation, diarrhea, etc.) were excluded. In addition, the exclusion criteria also included having used antibiotics, probiotics, or other nutritional supplements in the past three months. Feces collection tools and instructions were provided to each individual after a simple training. After the tubes with samples were submitted to workers, they were quickly frozen in liquid nitrogen and transferred to labs for further experimentation. This study was approved by the Human Ethics Committee of the Xi’an Jiaotong University. All subjects provided written informed consent.

### 2.2. Fecal Sample Preparation

A total of 5 mg feces was thawed and dispersed in 25 μL water which contains a mixture of methanol and internal standard. The mixture was then homogenized with zirconium oxide beads. After the homogenized suspension was centrifuged, the supernatant was transferred to a 96-well plate. The derivatization process was performed at 30 °C for 60 min on the sealed plate. The sample was diluted with ice-cold methanol solution (50%) and stored at −20 °C for 20 min. After centrifugation, the supernatant was then transferred to a plate with 10 μL internal standards in each well for further analysis.

### 2.3. Untargeted Metabolomics Analysis

Metabolic profiles were acquired on the platform of UltiMate 3000 HPLC (Dionex, Sunnyvale, CA, USA)-tandem Q Exactive Orbitrap MS (Thermo Fisher Scientific, Waltham, MA, USA). A column of Acquity UPLC HSS T3 (100 mm × 2.1 mm, 1.7 μm, Waters, Milford, MA, USA) was used for chromatographic separation. The mobile phase consisted of water containing 0.1% formic acid (mobile phase A) and solvent acetonitrile (mobile phase B). The gradient conditions were as follows: 0–1.0 min, 5% B; 1.0–9.0 min, 5~100% B; 9.0–12.0 min, 100% B; and 12.0–15.0 min 5% B. The sample injection volume was 5 μL with a flow rate of 0.35 mL/min. The column temperature was set at 40 °C. MS acquisition was as the below, scan type of 80–1200 *m*/*z*, resolution of 70,000, sheath gas flow rate of 38 (negative)–40 (positive) arb. QC samples were made by pooling and mixing a part of volume of each sample. The QC samples were injected at intervals of six samples to evaluate the stability and reproducibility of the analytical instruments.

UHPLC-MS data were analyzed by Compound Discoverer 2.1 (Thermo Fisher Scientific, Waltham, MA, USA). Peak extraction, peak alignment, peak correction, and standardization processes were performed to the raw data. A table was formed with mass and retention time pairs with associated intensities for all detected peaks. Then the metabolic results of positive and negative ions were combined for subsequent analysis.

### 2.4. Statistical Analysis of Metabolic Profiling Data

The processed tables were put into the MetaboAnalyst platform (www.metaboanalyst.ca (accessed on 24 February 2021)) for the multivariate statistical analysis. A partial least squares-discriminant analysis (PLS-DA) model was constructed for feature selector and classifier. Orthogonal partial least squares-discriminant analysis (OPLS-DA) was also used to improve the group separation. The robustness and validity of the OPLS-DA model were tested using the 2000-permutation test. The variable importance in projection (VIP) coefficient calculated based on the PLS-DA model was used for subsequent differential metabolite identification. VIP coefficient reflects the importance of a certain metabolite in discriminating groups and the metabolites with higher VIP values are more important in providing group separation. Meanwhile, the fold change (FC) in concentrations between groups were also calculated. The Mann–Whitney U test was used to analyze the significance of the metabolite differences between the groups. The screening criteria of differential metabolites were set as VIP value > 1 and *p* value < 0.05 in this study. Besides KBD vs. control, we also identified the differential metabolites between Grade I KBD vs. control, Grade II KBD vs. control, and Grade II KBD vs. Grade I KBD. A pathway analysis of differential metabolites was then performed on IMPaLA platform (http://impala.molgen.mpg.de/ (accessed on 13 July 2023)). An interaction network was constructed by MetaboAnalyst for the differential metabolites within certain significant pathways.

## 3. Results

### 3.1. Characteristics of the Study Subjects

After applying the inclusion and exclusion criteria, 75 subjects including 46 KBD patients and 29 healthy controls were enrolled in this study. The basic characteristics of the study subjects were summarized in Table 1. As the table showed, there were 36 males (23 for KBD and 13 for control) and 39 females (23 for KBD and 16 for control). The average age was 58.99 ± 7.43 in KBD patients and 57.54 ± 8.22 in control group. Among the KBD, there are 19 Grade I patients and 27 Grade II patients.

### 3.2. The Differential Metabolites between KBD and Control

**Figure 1:**
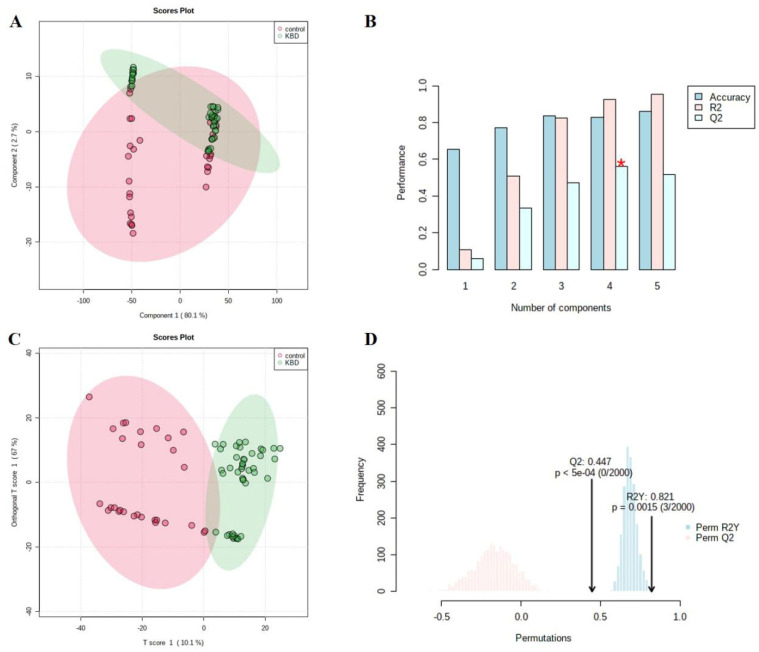
Multivariate statistical analysis results of PLS-DA and OPLS-DA: (**A**) PLS-DA score plots of KBD and control groups; (**B**) validation of the PLS-DA model by cross validation, under different number of components. The red star indicates the best classifier; (**C**) OPLS-DA score plot of KBD and control groups; and (**D**) OPLS-DA model validation with permutation tests (2000 permutations *p* < 0.01) and cross validation (R2Y = 0.821 and Q2 = 0.441).

**Figure 2:**
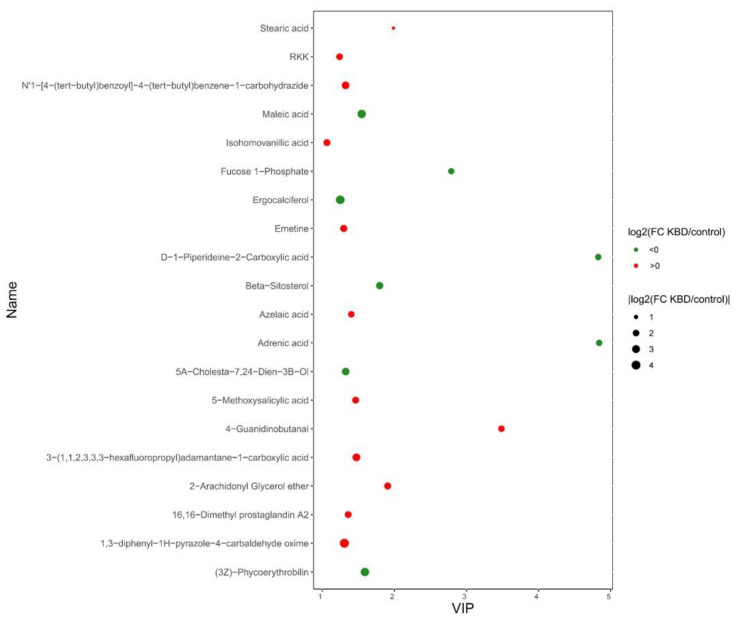
The scatter plot of the differential metabolites with the top 20 VIP value between KBD vs. control. X indicates the value of VIP. The higher VIP a metabolite has, the more contribution it provides in the group separation. The color indicates the upregulation (log_2_FC > 0) and downregulation (log_2_FC < 0). The size of the spot indicates the absolute value of log_2_FC.

Firstly, the multivariate statistical analyses were conducted, including partial least squares-discriminant analysis (PLS-DA) and orthogonal partial least squares-discriminant analysis (OPLS-DA). As the PLS-DA results show (Figure 1A), KBD samples clustered in a small zone while the controls scattered in a larger zone. Under the OPLS-DA model (Figure 1C), KBD and controls distributed in two distinguished clusters. After screening by the criteria of VIP > 1 and *p* < 0.05, a total of 584 differential metabolites were identified including 127 with lower levels in KBD and 457 with higher levels (Table S1), such as adrenic acid (log_2_FC = −1.87, VIP = 4.84, *p* = 7.63 × 10^−7^), hydrogen phosphate (log_2_FC = −2.57, VIP = 1.27, *p* = 1.02 × 10^−3^), taurochenodeoxycholic acid (VIP = 1.16, log_2_FC = −3.24, *p* = 0.03), prostaglandin E3 (VIP = 1.17, log_2_FC = 2.67, *p* = 5.61 × 10^−4^), prostaglandin A2 (VIP = 1.02, log_2_FC = 1.52, *p* = 2.68 × 10^−3^), etc. The metabolites with top 20 VIP value were summarized and exhibited in Figure 2. Detailed information of all differential metabolites can be seen in Table S1.

### 3.3. Pathway Analysis of Differential Metabolites

**Figure 3:**
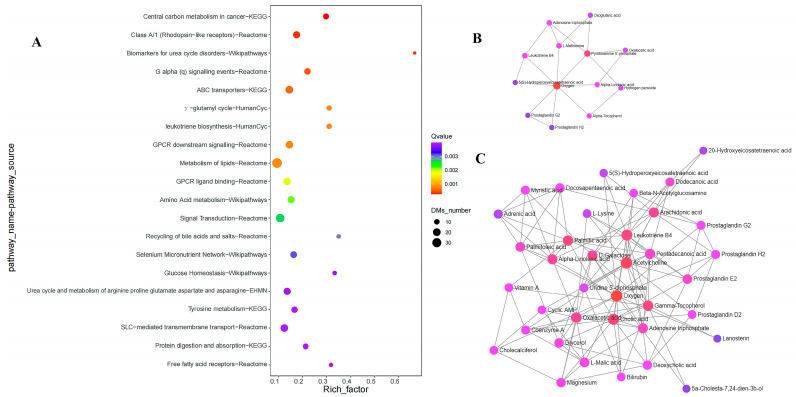
The results of pathway analysis (**A**) and the interaction of differential metabolites (**B**,**C**). (**A**) shows the top 20 smallest Q-value pathways (all Q value < 0.05), (**B**) shows the interaction of differential metabolites from the significant pathway of selenium micronutrient network (Wikipathways), and (**C**) shows the interaction of differential metabolites from significant pathways of metabolism of lipids (Reactome) and free fatty acid receptors (Reactome).

We then conducted pathway analysis for differential metabolites to further explore the functional implications of the above results. As the results showed (Figure 3 and Table 2), the compounds were primarily involved in the pathways such as selenium micronutrient network (Q value = 3.11 × 10^−3^, Wikipathways), metabolism of lipids (Q value = 8.43 × 10^−4^, Reactome), free fatty acid receptors (Q value = 3.99 × 10^−3^, Reactome), recycling of bile acids and salts (Q value = 2.98 × 10^−3^, Reactome), protein digestion and absorption (Q value = 3.93 × 10^−3^, KEGG), and amino acid metabolism (Q value = 2.17 × 10^−3^, Wikipathways). The interaction network of compounds were constructed based on the differential metabolites from selenium-related and lipid-related significant pathways of (Figure 3B,C).

A total of 14 differential metabolites were found to be related to the Selenium micronutrient network (Wikipathways), which involves the most relevant biochemical processes related to selenium. The involved metabolites included prostaglandin E3 (VIP = 1.17, log_2_ (FC_KBD*vs*C_) = 2.67, *p* = 5.61 × 10^−4^), prostaglandin G2 (VIP = 1.08, log_2_ (FC_KBD*vs*C_) = −1.91, *p* = 3.15 × 10^−3^), 3,3,5−Triiodo−L−Thyronine (VIP = 1.17, log_2_(FC_KBD*vs*C_) = −1.47, *p* = 4.97 × 10^−3^), L-Methionine (VIP = 1.02, log_2_ (FC_KBD*vs*C_) = −1.92, *p* = 7.31 × 10^−3^), L-Methionine S-Oxide (VIP = 1.09, log_2_ (FC_KBD*vs*C_) = −1.43, *p* = 5.25 × 10^−3^), etc. The interactions between the metabolites are exhibited in Figure 3B. The metabolites involved in each pathway are summarized in Table S2.

### 3.4. The Results of Comparisons between Subgroups

**Figure 4:**
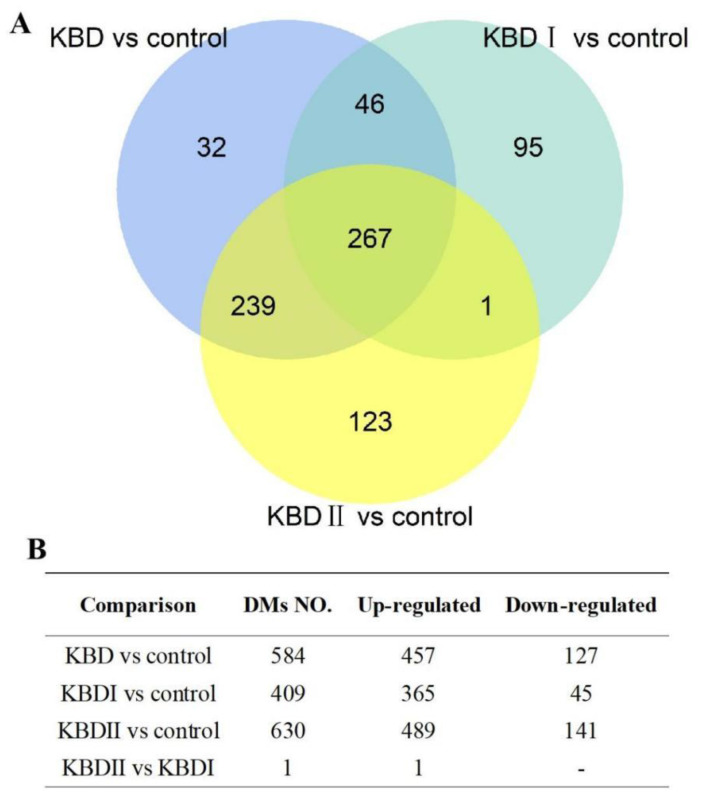
The summary of results in different comparisons: (**A**) shows the Venn plot of differential metabolites number from KBD vs. control, KBD II vs. control, and KBD I vs. control as well as their overlapping and (**B**) summarizes the number of differential metabolites from KBD vs. control, KBD II vs. control, KBD I vs. control, and KBD II vs. KBD I.

Besides the comparison of KBD vs. control, we also detected the differential metabolites between subgroups, including KBD II vs. control, KBD I vs. control, and KBD II vs. KBD I. The results are summarized in Figure 4. In the results, there are 630 differential metabolites between KBD II vs. control, while 409 between KBD II vs. control and only 1 differential metabolite between KBD II vs. KBD I. The Venn plot showed the overlap of differential metabolites between the results of different comparisons (Figure 4A). In the results, a total of 267 differential metabolites were found to be shared by KBD vs. control, KBD II vs. control and KBD I vs. control. Detailed information about the overlapped metabolites is summarized in Table S3.

## 4. Discussion

In summary, we conducted a fecal sample metabolomics study for Kashin–Beck disease, an endemic osteoarthropathy of China with unclear pathogenesis, to investigate the potential intermediary role of intestinal metabolites in the progression of KBD damage. We identified a group of differentially regulated metabolites in KBD compared with the control group, which might be involved in the etiology and pathogenesis of KBD. Using pathway analysis, these differential metabolites were found to be functionally related to selenium-related biochemical process, lipid metabolism, bile acid metabolism, amino acid metabolism, etc.

Adrenic acid was one of the differential metabolites between the KBD and control with the top VIP value (4.84) and was significantly decreased in KBD feces (log_2_FC = −1.87). It is a long-chain polyunsaturated free fatty acid consisting of 22 carbon atoms and is formed through a 2-carbon chain elongation of arachidonic acid (20:4n6) (AA) by elongase [17]. Previous research showed that adrenic acid had potent anti-inflammatory effects [18]. Specifically, it could efficiently inhibit chemoattractant leukotriene B4 (LTB4) production as well as its precursor and reduced further attraction of neutrophils to the site of inflammation [18]. In vivo treatment of adrenic acid markedly prevented inflammation and alleviated the arthritis symptoms in an LTB4dependent murine arthritis model [18]. Another study demonstrated that intestinal metabolic disturbance occurred in a post-traumatic osteoarthritic (PTOA) mouse model with feces adrenic acid significantly decreased [19]. Prebiotic treatment could exert a beneficial influence on the joint degeneration of PTOA mice, possibly through compensating for such a decrease [19]. Furthermore, adrenic acid was found to be highly correlated with femur and tibia bone mineral density (BMD) [20]. All this evidence supported the important role of adrenic acid in the bones and cartilage health, which was possibly due to its anti-inflammatory effects. According to previous studies, both inflammation and osteoporosis were prominent in adult KBD patients [21,22,23]. For example, excessive expression of several pro-inflammatory compounds, such as IL-1 and IL-6, was seen in both KBD serum and KBD cartilage, as well as nitric oxide in KBD serum [21,22]. All the above clues suggest that adrenic acid might become involved in the pathogenesis of KBD partly through its regulation in inflammation and BMD. However, further investigations of in vivo and in vitro designs are still needed to verify the hypothesis.

Hydrogen phosphate was another differential compound (decreased significantly in KBD with log_2_FC = −1.87) that was closely related to the bone and cartilage homeostasis. Phosphorus is the second most abundant mineral in the human body and is mainly stored in bone (85%), by conjugating with calcium in hydroxyapatite crystals deposited onto the collagen matrix [24]. Biomineralization processes in bone included the transformation of an amorphous phosphate-based precursor to highly organized nanocrystals [25]. Hydrogen phosphate or ionic phosphate species, as the simplest type of inorganic phosphate, are the fundamental building blocks of biological minerals [25]. Besides this structural role, current opinion also regards them as a regulatory factor in multiple bone metabolism processes such as osteoblastic proliferation, bone resorption, and chondrocyte differentiation [24]. The interactive regulatory elements with them included signaling pathways (ERK1/2), transcription factors (Fra-1, Runx2), and phosphate transporters (PiT1, PiT2) [24]. KBD initially occurs in the developing skeleton, where active endochondral ossification happens and then exhibits prominent osteoarthritis phenotypes [26]. Our results indicated that a disturbance of phosphate metabolism existing in KBD was possibly related to its bone development failure [26].

Among the differential metabolites, bilirubin exhibited notably decreased level in KBD patients with a log_2_FC of −6.82. Bilirubin is a constituent of the bile that is formed as a breakdown product of hemoglobin. Generally, a low level of it is not seen to be a major concern itself. However, certain medications can decrease the bilirubin levels. In this scenario of KBD patients, a history of non-steroidal anti-inflammatory drugs (NSAIDs) use is the most possible explanation for this finding, since the problem of painful joints are prominent in KBD. Besides bilirubin, morphine was surprisingly found to be elevated in KBD patients (log_2_FC = 1.74). But in our questionnaire survey, no one declared the use of morphine-containing medicines. We speculated it might be from the use of unknown herbs by KBD patients, which could contain morphine. From our observation, after being disappointed by common pain-relief medicines, KBD patients may resort to folk medicines, which are usually made of various herbs. Such folk medicines have not been officially verified or approved; this could increase the risk of exposure to compounds such as morphine. The above evidence reminds us that the abuse of medicines in KBD regions is beyond imagination and is worthy of more concerns. Additionally, we should be aware that elevated morphine could have an impact on the metabolomics profile beyond KBD.

Further pathway analysis results revealed that several differential metabolites were involved in the recycling of bile acids and salts (Reactome); this suggested that there was a perturbation in the bile acid homeostasis. Bile acids (BA) are a group of small amphipathic molecules which were mainly synthesized in liver, secreted into the intestines, and transformed to secondary types by gut microbiota. They could also be reused by the gut–liver axis. The balance of this axis is essential for multiple physiology functions, such as metabolic homeostasis (especially lipid metabolism), gut barrier integrity, and gene expression [27]. Recent studies also revealed that compounds in this pathway were closely related to bone and cartilage integrity [28,29]. For example, taurochenodeoxycholic acid, by conjugation of chenodeoxycholate with taurine, is a kind of primary BA in this pathway, with a significantly lower level in KBD patients. It was identified by previous research to have a protective effect in endoplasmic reticulum (ER) stress-induced chondrocyte apoptosis [30]. Another study found that it could increase the bone mineral density and mice distal femur in mice model [31]. Chenodeoxycholic acid, another differential compound in this pathway, was also proven to be able to markedly reduce the release of matrix metalloproteinase in osteoarthritis [32]. Our results suggested that compounds in bile acid might participate in the progression of KBD. However, the exact mechanism remains to be elucidated.

Using pathway analysis, we also found that a group of differential metabolites in KBD was involved in the selenium micronutrient network (Wikipathways, https://www.wikipathways.org/pathways/WP15.html, http://impala.molgen.mpg.de/ (accessed on 13 July 2023)), which indicates the biochemical processes related to selenium. The close relationship of selenium and KBD has been demonstrated by plenty of studies [2]. The initial clue is from the fact that KBD area in China largely overlapped the selenium-deficiency geographical belt, which leads a lower level of selenium in local water, soil, cereal, and corn [2]. The following population investigations demonstrated an overall decrease in selenium level in KBD patients’ serum and hair [3,4,5]. Our results further suggest that metabolic processes in the intestines might also be affected by a selenium-deficient diet and that the dysregulated metabolites were possible participators in selenium to KBD damage cascade. Selenium is an essential trace element whose role was played by constituting selenoprotein, which further participates in various physiological functions such as redox homeostatic, anti-inflammation, and thyroid hormone processing. Several prostaglandins, which were involved in this pathway, were found to be dysregulated in KBD, such as prostaglandin E3, prostaglandin A2, prostaglandin G2, prostaglandin F2A, prostaglandin H2, etc. Synthesis of prostaglandins from arachidonic acid relied on the oxidation of cyclooxygenases, which can be inhibited by the antioxidant defense from selenium-containing enzymes, glutathione peroxidase (mainly GPX4 and GPX2) [33]. Prostaglandins work as mediators to increase the production of matrix metalloproteinases by chondrocytes and are the treatment target of joint pain in osteoarthritis [34,35]. The results suggest an overall dysregulation of prostaglandins in KBD, which was part of the selenium-centered metabolism system. Until now, few studies have focused on the role of prostaglandins in KBD. Additionally, reverse triiodothyronine (or 3,3,5-Triiodo-L-Thyronine, C07639) is another disturbed compound in this pathway with lower level in KBD. It is a metabolically inactive form of thyroid hormone and generated from T4 by the type 3 5′-deiodinase enzyme, a kind of selenoprotein. Previous investigation found the blood T3 level decreased significantly in preschool children in KBD-area [36]. But another study did not find such a difference in T3 level but instead observed a lower level of T4 in KBD [37]. Transcriptome and genetic integrative study identified a close link between DIO2, which encode type 2 deiodinase containing the selenium and KBD [26,38]. Our results also suggested a potential disturbance in thyroid hormone metabolism. However, there is a lack of a systematic study exploring the complex interaction of selenium and thyroid hormone production in KBD. Further studies are still warranted to explore the role of selenium and its interactions with other molecules in KBD progression.

The pathway analysis also indicated a disrupted lipid metabolism occurring in KBD, which is consistent with previous metabolomics study results conducted in KBD serum [14]. The involved pathways included metabolism of lipids (Reactome) and free fatty acid receptors (Reactome). In addition, the bile acid’s metabolism discussed above was closely related to lipid metabolism. Lipids, comprising three main types of phospholipids, sterols, and triglycerides, work under a complicated regulatory system and play essential roles in many physiological processes, including constructing cellular membranes, signal transduction, energy metabolism, and as precursors of inflammatory cytokines. The proper functioning of lipids and their metabolic components exerts significant influence on cartilage integrity by diverse ways [39]. For example, a previous study revealed that a high level of saturated fatty acids (SFAs) was directly associated with the progression of cartilage degradation through metabolic not mechanical methods [40]. Stearic acid, a kind of saturated fatty acid, was identified with a higher level in KBD fecal material in our study and was found to be able to induce proinflammatory cytokine production in chondrocytes partly through activation of the lactate-HIF1α pathway [41]. In addition, phospholipids and sterols were also found to have a role in bone or cartilage function (phospholipids with initial formation of calcium hydroxyapatite crystals during mineralization and cholesterol metabolic pathway as a regulatory role in chondrogenic differentiation during growth plate development [42,43]). These two types were also involved in lipid metabolism dysregulation of KBD, with lower glycerophosphocholine and cholesterol levels in KBD. Our results suggested an overall dysregulation of lipids in KBD intestines and might be involved in KBD development. However, further evidence is still needed to clarify the exact involved lipid type and the specific pathways.

In addition, according to the PLS-DA results, the KBD and control did not separate totally in the plot although the KBD subjects clustered well (but further OPLS-DA separated them clearly). Such results suggested that there might be some other features besides the metabolome profile existing in KBD. Previous studies found that KBD progression had a genetic component involved with a 40% heritability approximately [44]. We hypothesize that the identified KBD-related metabolites in this study will be regulated by a complicated regulatory system in the body before they finally exert the influence on target tissue and that the regulatory pattern will be highly associated with the genetic background of the KBD patients. In this case, omics design would likely be used for KBD study in the future.

There are also some limitations in this study. Firstly, this is a cross-sectional observational study. Although we applied a strict series of inclusion and exclusion criteria, we could not eliminate all the potential confounding factors. Additionally, it is important to be cautious when inferring a cause–effect relationship between metabolites and KBD damage. More multilevel studies are needed to further explore and specify the KBD metabolic signature and the underlying mechanisms. Secondly, due to the lack of blood samples, we did not measure the correlation between fecal metabolites and serum ones. However, the combined results of the two would be more informative (the former is related to the exposure and intestinal microenvironment more while the latter is related to the inner physiological function more).

We conducted a fecal metabolomics study in the KBD endemic area to identify the metabolic signature of KBD patients. As the results showed, we identified a group of differentially expressed metabolites, which might be associated with the initiation and development of KBD. Further pathway analysis suggested that there was a disturbance in lipid metabolism, bile acids, and salt recycling, as well as the selenium-related bioprocesses. Our study results provided new clues for the understanding of KBD etiology and pathogenesis. Linking the nutrients metabolism (especially selenium and lipid) to KBD cartilage damage should be a future direction for KBD study.

## Figures and Tables

**Table 1:** The characteristics of study population.

Demographic Factors	KBD	Control	*p*
Age	58.99 ± 7.43	57.54 ± 8.22	0.443
Sex (Male/Female)	23/23	13/16	0.813
BMI (kg/m^2^)	23.68 ± 3.95	23.77 ± 3.62	0.926
KBD Grade (Grade I/Grade II)	19/27	-	-
total	46	29	-

**Table 2:** The results of pathway analysis for differential metabolites (top 20 with Q value < 0.05).

Pathway Name	Pathway Source	DMs NO.	Metabolites NO. in Pathway	*p* Value	Q Value
Central carbon metabolism in cancer	KEGG	11	37	2.06 × 10^−7^	2.76 × 10^−4^
Biomarkers for urea cycle disorders	Wikipathways	6	9	4.21 × 10^−7^	3.76 × 10^−4^
Class A/1 (Rhodopsin-like receptors)	Reactome	17	97	4.68 × 10^−7^	3.76 × 10^−4^
G alpha (q) signalling events	Reactome	13	59	7.38 × 10^−7^	4.95 × 10^−4^
ABC transporters	KEGG	20	138	1.07 × 10^−6^	6.14 × 10^−4^
γ-glutamyl cycle	HumanCyc	9	29	1.85 × 10^−6^	8.05 × 10^−4^
leukotriene biosynthesis	HumanCyc	9	29	1.85 × 10^−6^	8.05 × 10^−4^
GPCR downstream signalling	Reactome	19	131	2.00 × 10^−6^	8.05 × 10^−4^
Metabolism of lipids	Reactome	37	394	2.30 × 10^−6^	8.43 × 10^−4^
GPCR ligand binding	Reactome	19	140	5.52 × 10^−6^	1.85 × 10^−3^
Amino Acid metabolism	Wikipathways	16	105	7.02 × 10^−6^	2.17 × 10^−3^
Signal Transduction	Reactome	26	243	9.22 × 10^−6^	2.65 × 10^−3^
Recycling of bile acids and salts	Reactome	7	20	1.11 × 10^−5^	2.98 × 10^−3^
Selenium Micronutrient Network	Wikipathways	14	86	1.24 × 10^−5^	3.11 × 10^−3^
Glucose Homeostasis	Wikipathways	7	21	1.61 × 10^−5^	3.81 × 10^−3^
Urea cycle and metabolism of arginine_ proline_ glutamate_ aspartate and asparagine	EHMN	17	125	1.72 × 10^−5^	3.85 × 10^−3^
Tyrosine metabolism	KEGG	13	78	1.97 × 10^−5^	3.93 × 10^−3^
SLC-mediated transmembrane transport	Reactome	19	153	2.05 × 10^−5^	3.93 × 10^−3^
Protein digestion and absorption	KEGG	10	47	2.05 × 10^−5^	3.93 × 10^−3^
Free fatty acid receptors	Reactome	7	22	2.28 × 10^−5^	3.99 × 10^−3^

Note: DMs, differential metabolites.

## Data Availability

Data is contained within the article or Supplementary Material.

## Supplementary Materials

The following supporting information can be downloaded at: https://www.mdpi.com/article/10.3390/nu15214651/s1, Table S1: differential metabolites between KBD vs. control; Table S2: differential metabolites in significant pathways (Top 20 smallest Q-value pathways); and Table S3: the shared differential metabolites among results of KBD vs. control, KBD II vs. control, and KBD I vs. control.

## Abbreviations

KBD	Kashin–Beck disease
DM	differential metabolites
PLS-DA	partial least squares-discriminant analysis
OPLS-DA	orthogonal partial least squares-discriminant analysis
BAs	bile acids
T3	triiodothyronine
T4	thyroxine
